# Novel Pharmacological Activity of Artesunate and Artemisinin: Their Potential as Anti-Tubercular Agents

**DOI:** 10.3390/jcm6030030

**Published:** 2017-03-10

**Authors:** Won Hyung Choi

**Affiliations:** 1 Department of Biomedical Science, Kyung Hee University School of Medicine, 26 Kyunghee-daero, Dongdaemun-gu, Seoul 02447, Korea; whchoi@khu.ac.kr; Tel.: +82-10-2071-5679; Fax: +82-70-8269-5679; 2 Department of Medical Zoology, Kyung Hee University School of Medicine, 26 Kyunghee-daero, Dongdaemun-gu, Seoul 02447, Korea

**Keywords:** artesunate, isoniazid, MGIT 960 system, Ogawa slant medium, susceptibility-test

## Abstract

Tuberculosis is a major infectious disease that globally causes the highest human mortality. From this aspect, this study was carried out to evaluate novel pharmacological activities/effects of artesunate and artemisinin causing anti-tubercular activity/effects against *Mycobacterium tuberculosis* (Mtb). The anti-Mtb activities/effects of artesunate and artemisinin were evaluated using different anti-Mtb indicator assays, such as the resazurin microtiter assay, the Mycobacteria Growth Indicator Tube (MGIT) 960 system assay, and the Ogawa slant medium assay, as well as in vivo tests. Artesunate showed selective anti-Mtb effects by strongly inhibiting the growth of Mtb compared to artemisinin, and consistently induced anti-Mtb activity/effects by effectively inhibiting Mtb in the MGIT 960 system and in Ogawa slant medium for 21 days with a single dose; its minimum inhibitory concentration was 300 µg/mL in in vitro testing. Furthermore, artesunate demonstrated an anti-tubercular effect/action with a daily dose of 3.5 mg/kg in an in vivo test for four weeks, which did not indicate or induce toxicity and side effects. These results demonstrate that artesunate effectively inhibits the growth and/or proliferation of Mtb through novel pharmacological activities/actions, as well as induces anti-Mtb activity. This study shows its potential as a potent candidate agent for developing new anti-tuberculosis drugs of an effective/safe next generation, and suggests novel insights into its effective use by repurposing existing drugs through new pharmacological activity/effects as one of the substantive alternatives for inhibiting tuberculosis.

## 1. Introduction

*Mycobacterium tuberculosis* (Mtb) is one of the most dangerous pathogens which cause the highest human mortality and morbidity among various infectious diseases worldwide, particularly in Africa and the developing countries including Southeast Asia. Tuberculosis (TB) indicated a high level of co-infection with HIV (human immunodeficiency virus) from 2004 to 2014, and the prevalence of TB cases co-infected with HIV was highest in the African region compared with other regions [1,2]. For these reasons, various anti-TB drugs, such as rifampicin, isoniazid, ethambutol, pyrazinamide, and second-line drugs (D-cycloserine, cephalosporin, viomycin, etc.), were developed for the treatment of TB patients. Furthermore, various studies and strategies on tuberculosis, which causes serious complications to patients and resistance to existing agents, have been carried out globally. 

Recently, new compounds, such as PA-824, SQ-109, linezolid, bedaquiline, ripafentine, moxifloxacin, and gatifloxacin, have been consistently developed as new/repurposed anti-TB drugs through chemical tailoring and remodeling of the existing antibacterial drugs, which are presently being tested in Phase II or Phase III trials [1,2,3,4]. Furthermore, compounds such as pyridomycin, thiophenes, and Q203 have been developed as novel anti-TB drugs [5,6,7]. In spite of these efforts and anti-TB drug developments, the rapid emergence of TB and multidrug-resistant TB (MDR-TB) or extensively drug-resistant TB (XDR-TB) has consistently worsened the public health problem worldwide. Until recently, medicinal plants used in traditional oriental medicine were utilized as traditional medical resources for treating chronic diseases and/or symptoms which are induced by viral and parasitic infections, hepatitis, and cancer, in both “in vivo” and “in vitro” [8,9,10,11]. Furthermore, recently it was reported that various extracts and/or natural products derived from medicinal plants or traditional oriental medicines significantly indicated anti-Mtb activities/effects against Mtb [12,13,14,15,16]. Thus, effective new anti-TB drugs with novel mechanisms of action, low cytotoxicity, and safety are urgently needed to inhibit and to combat the global tuberculosis epidemic. From these perspectives, artemisinin is an anti-malaria first-line drug derived from the plant *Artemisia annua*, and artesunate is a semi-synthetic derivative of artemisinin, a part of the artemisinin group. In addition, they are being effectively used as anti-malaria drugs. For this reason, this study started from the hypothesis that artesunate and artemisinin may effectively modulate or inhibit the growth/proliferation of Mtb causing tuberculosis. The main objective of this study was carried out to evaluate the anti-Mtb activities/effects of artesunate and artemisinin which are effectively used as anti-malaria first-line drugs, and to determine their potential as promising candidate drugs for developing novel anti-TB drugs for a safe/effective next generation.

## 2. Experimental Section

### 2.1. Materials

Various materials used in this study, rifampicin, isoniazid, ethambutol, artesunate, artemisinin, resazurin powder, and DMSO, were purchased from Sigma-Aldrich Chemical, Co., Ltd. (St. Louis, MO, USA), and MGIT™ 960 system indicator 7 mL tubes with BACTEC™ MGIT™ 960 supplement kit, and 7H11 agar were purchased from Becton-Dickinson and Company (Sparks, MD, USA). 2% Ogawa slant medium was purchased from Union Lab Co., Ltd. (Seoul, Korea). All other chemicals and reagents were purchased from Merck Chemical Co., Ltd. (Darmstadt, Germany) and Sigma-Aldrich Chemical Co., Ltd. (St. Louis, MO, USA). 

### 2.2. Animals

Rats (six weeks, female/Sprague Dawley) were purchased from DaeHan Bio-Link Co., Ltd. (Chungcheongbuk-do, Korea), and all animals were kept at 23 ± 0.5 °C and in a 12 h light/dark cycle in a controlled environment of the central animal care facility. Food and water were provided ad libitum to all animals. All experimental animals and the facility used for this study were strictly maintained in accordance with the guidelines of the National Institutes of Health for the Care and Use of Laboratory Animals, and the Kyung Hee University IACUC.

### 2.3. Preparation of Anti-MTB Drugs

The anti-Mtb first-line drugs, isoniazid and ethambutol, were dissolved in sterile distilled water, and rifampicin was dissolved in DMSO, to a concentration of 50 mg/mL according to the manufacturer’s instruction. The anti-Mtb first-line drugs were used as reference standard drugs. All compounds were filtered using 0.2 μm membrane syringe filters (Roshi Kaisha, Ltd., Tokyo, Japan) before use, and were stored at −80 °C deep-freezer until use. 

### 2.4. Preparation and Culture Conditions of M. tuberculosis

*Mycobacterium tuberculosis* H37R_V_ (ATCC 27294) used in this study was purchased from American Type Culture Collection (Manassas, VA, USA). Mtb H37R_V_ was grown in Middlebrook 7H9 broth (Difco Laboratories, Detroit, MI, USA) supplemented with 10% (*v*/*v*) oleic acid/albumin/dextrose/catalase (OADC) enrichment (Becton, Dickinson and Company, Sparks, MD, USA) and 0.05% (*v*/*v*) Tween 80 (Sigma-Aldrich Chemical, St. Louis, MO, USA) at 37 °C for five weeks with a shaking incubation of 100 rpm until the mid-log phase. 

### 2.5. Determination of Drug Susceptibility of M. tuberculosis

The in vitro anti-Mtb activity of artesunate and artemisinin against *M. tuberculosis* was confirmed by a resazurin microtiter assay (REMA) using a 96-well micro-plate. Briefly, Mtb was grown in fresh Middlebrook 7H9 broth supplemented with 10% (*v*/*v*) oleic acid/albumin/dextrose/catalase (OADC) enrichment and 0.05% (*v*/*v*) Tween 80 until the culture reached a turbidity equal to that of 1.0 McFarland standard (3.0 × 10^8^ CFU/mL) at 37 °C. The bacteria were adjusted to a density of 2 × 10^6^ CFU/mL in fresh culture broth. Finally, the bacterial suspensions were inoculated into all wells of a 96 well microtiter plate containing final concentrations (9.375–300 µg/mL) of artesunate, artemisinin, and anti-Mtb first-line drugs (1.25–5 µg/mL), and growth controls containing no anti-Mtb first-line drugs and blank controls without inoculation were also included. The 96 well-plates, covered with lids, placed in a plastic bag, were incubated at 37 °C for five days. After incubation, 20 μL of freshly prepared 0.05% (*v*/*v*) resazurin solution was added to all wells of a 96 well microtiter plate, and the plates were re-incubated at 37 °C for two days. A change in color from blue to pink indicating bacterial growth showed the reduction of resazurin caused by bacterial metabolism, which was observed after two days of incubation. The MIC (minimum inhibitory concentration) was expressed as the lowest concentration of the drug that inhibited Mtb-growth or prevented change in color of the resazurin from blue to pink based on a REMA. 

### 2.6. Evaluation of Drug Susceptibility of M. tuberculosis by MGIT 960 System Assay

To evaluate the anti-Mtb effects of artesunate and artemisinin against the growth/proliferation of *M. tuberculosis*, drug susceptibility testing (DST) of the strain was performed using the BACTEC™ MGIT 960 system (Becton, Dickinson and Company, Sparks, MD, USA). In brief, 100 μL of a suspension of *M. tuberculosis* culture, adjusted to 9.6 × 10^6^ CFU/mL, was inoculated in an MGIT growth media tube with BACTEC™ MGIT 960 growth supplement (Becton, Dickinson and Company) and the compounds, which was incubated into the BACTEC™ MGIT 960 system for three weeks. 

### 2.7. Drug Susceptibility Test of M. tuberculosis by Ogawa Slant Medium Assay

The anti-Mtb effects of artesunate and artemisinin against *M. tuberculosis* were evaluated by 2% Ogawa slant medium assay. In brief, the bacteria were adjusted to a density of 2 × 10^6^ CFU/mL in fresh Middlebrook 7H9 broth supplemented with 10% (*v*/*v*) oleic acid/ albumin/dextrose/catalase (OADC) enrichment (Becton-Dickinson and Company, Sparks, MD, USA) and 0.05% (*v*/*v*) Tween 80. The bacterial suspensions were inoculated into all wells of a 96 well microtiter plate containing artesunate, artemisinin, anti-Mtb first-line drugs, and growth controls containing no anti-Mtb first-line drugs, respectively. Blank controls without inoculation were also included. The 96 well-plates, covered with lids, placed in a plastic bag, were incubated at 37 °C for five days. After five days of the incubation, 20 μL of a suspension of *M. tuberculosis* culture in 96 well-plates was inoculated on 2% Ogawa slant medium, and the Ogawa slant media were incubated at 37 °C for three weeks. 

### 2.8. Evaluation of In Vivo Anti-Mtb Effects of Artesunate and Artemisinin

**Figure 1:**
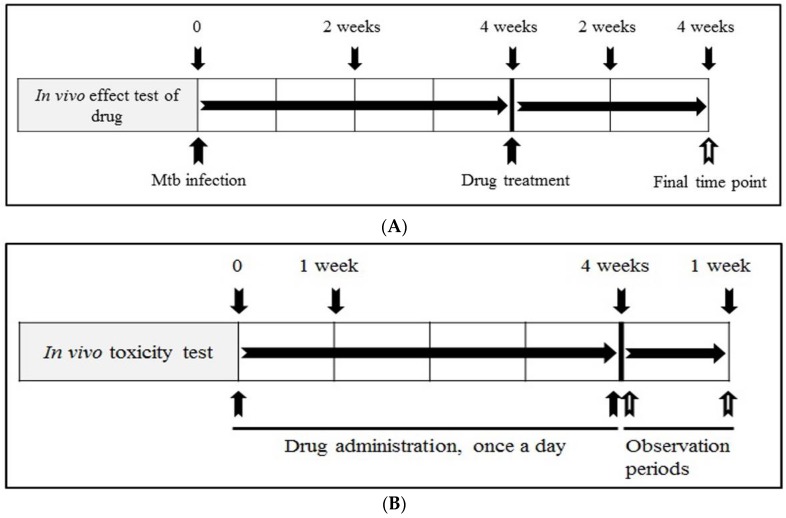
Experimental design for in vivo tests of artesunate and artemisinin. (**A**) The evaluation of in vivo effects; the rats were divided into a normal group (*n* = 5), positive control group (*n* = 5/isoniazid group) and experimental groups (artesunate and artemisinin groups, *n* = 5/each group). The rats were orally administered with a single dose of 5 mg/kg of isoniazid, and 3.5 mg/kg of artemisinin and artesunate, once a day for four weeks, after four weeks of Mtb infection, respectively. (**B**) The evaluation of in vivo toxicity; the rats were orally administered with two different doses (1.75 and 3.5 mg/kg) of artemisinin and artesunate, once a day for four weeks of the experiment, respectively, and additionally observed for seven days.

Rats were divided into a normal group (*n* = 5/uninfected group), positive control group (*n* = 5/isoniazid group) and experimental groups (artesunate and artemisinin groups, *n* = 5/each group). The rats were anaesthetized by intraperitoneal injection, and infected with 30 μL of a suspension of Mtb adjusted to a density of 2 × 10^5^ CFU through the intranasal route. In the experimental groups, the rats were treated with daily dosage of 5 mg/kg of isoniazid, and 3.5 mg/kg of artesunate and artemisinin by oral administration, once a day for four weeks, after four weeks of Mtb-infection, respectively. To determine the bacterial burden in the lungs, the animals were euthanized by CO_2_ gas in CO_2_ chamber at each time point. The lungs were immediately removed aseptically in a clean bench. The organs were homogenized in 1X PBS with 0.05% (*v*/*v*) Tween 80, and total viable count at each time point was measured by serial dilution plating on Meddlebrook 7H11 agar (Becton, Dickinson and Company, Franklin Lakes, NJ, USA). The experimental design of in vivo tests of the compounds is shown in Figure 1A, and the animals were carefully maintained in a central animal care facility during the experiment. 

### 2.9. Measurement of Body Weight and In Vivo Toxicity Test 

To evaluate the in vivo toxicity of artesunate and artemisinin, rats were divided into normal (*n* = 4) and experimental groups (artesunate and artemisinin groups, *n* = 4/each group). The two different doses (1.75 and 3.5 mg/kg) of artesunate and artemisinin was administered orally in rats of the experimental groups, once a day for four weeks, respectively, and the rats were additionally observed for seven days. The body weight of all animals was recorded at each time point, once a day for 28 days, and the experimental design of the in vivo toxicity-tests of the compounds is shown in Figure 1B. The animals were euthanized using CO_2_ gas in CO_2_ chamber at final time point after the recording of the body weight. The animals were carefully maintained in a central animal care facility during the experiment. 

### 2.10. Statistical Analysis

All results were expressed as mean ± standard deviation (S.D.) of three independent experiments. Statistical analysis of the data was performed using the Student’s *t*-test and one-way analysis of variance (ANOVA). * *p* < 0.05 was considered to be statistically significant.

## 3. Results 

### 3.1. Evaluation of Anti-Mtb Activity of Artesunate and Artemisinin against M. tuberculosis

**Figure 2:**
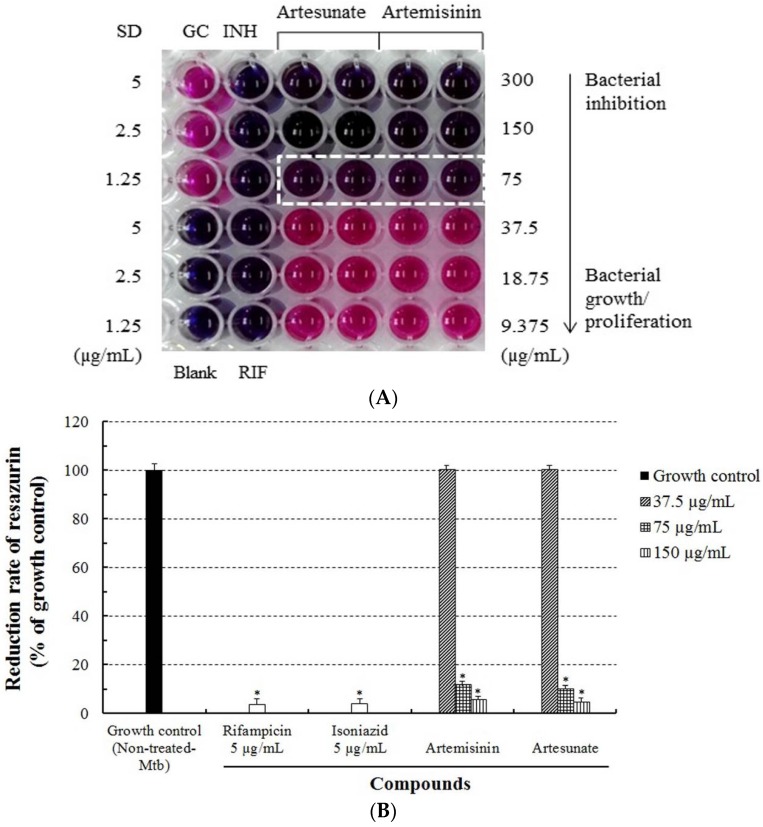
The in vitro anti-Mtb activity/effect of artesunate and artemisinin against Mtb H37Rv measured using the REMA. (**A**) The viability of Mtb determined by a change in color of the REMA; and (**B**) the quantification analysis of the REMA that indicates the Mtb inhibitory rate of the compounds. Mtb was incubated with different concentrations (9.375–300 µg/mL) of the compounds, respectively. The blue color and dotted lines show the anti-Mtb activity/effect, and the pink color indicates bacterial growth. (GC; growth controls containing no antibiotic, Blank; blank controls without inoculation, SD; standard reference drugs (INH; isoniazid, RIF; rifampicin, 1.25–5 µg/mL)). All results were expressed as mean ± standard deviation (S.D.) of three independent experiments. * *p* < 0.05 was considered to be statistically significant.

The anti-Mtb activities of artesunate and artemisinin were evaluated using the REMA. After the bacteria were incubated with various concentrations (9.375–300 µg/mL) of artesunate, artemisinin, and anti-Mtb first-line drugs (rifampicin and isoniazid) for five days, their viabilities were markedly inhibited in a concentration-dependent manner, and were 0% at a concentration of 75 µg/mL of artesunate and artemisinin (Table 1). Artesunate and artemisinin demonstrated their Mtb-inhibitory effects causing anti-Mtb activity through the bacterial color change observed in the REMA (Figure 2). In addition, the MIC values of both artesunate and artemisinin against the growth of Mtb were measured as 75 µg/mL, respectively (Table 2). However, artesunate did not indicate higher anti-Mtb activity/effects than artemisinin in the REMA. These results demonstrate that artesunate and artemisinin have not only anti-Mtb activity which strongly inhibits the growth/proliferation of Mtb, but also unique and selective anti-Mtb properties or functions. 

### 3.2. Anti-Mtb Effects of Artesunate and Artemisinin against the Growth/Proliferation of M. tuberculosis 

**Figure 3:**
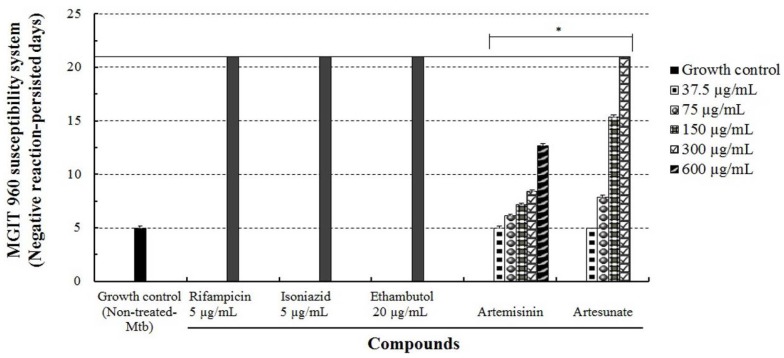
The anti-Mtb effect of artesunate and artemisinin measured using the MGIT 960 system assay. Mtb was incubated with different concentrations (37.5–600 µg/mL) of the compounds in the MGIT 960 system device at 37 °C for three weeks, respectively. All results were expressed as mean ± standard deviation (S.D.) of three independent experiments. * *p* < 0.05 was considered to be statistically significant.

The anti-Mtb effects of artesunate and artemisinin against *M. tuberculosis* were further evaluated using the MGIT 960 system assay. The bacteria were incubated with various concentrations (37.5–600 µg/mL) of artesunate or artemisinin and anti-Mtb first-line drugs (5 and 20 µg/mL) in an MGIT growth media tube of the BACTEC^TM^ MGIT 960 system device for drug susceptibility testing for three weeks, and their growth units were markedly inhibited in a concentration-dependent manner. Particularly, when Mtb was incubated with 150 µg/mL of artesunate, the growth unit of Mtb was detected at 15 days, whereas 300 µg/mL of artesunate strongly inhibited the growth of Mtb, and the growth unit of Mtb was not detected at 21 days (Figure 3). However, when Mtb was incubated with 600 µg/mL of artemisinin, their growth units were detected at about 12.6 days. In the MGIT 960 system assay, artesunate more effectively inhibited the growth of Mtb for three weeks compared to artemisinin, and its minimum inhibitory concentration (MIC) was measured as 300 µg/mL (Table 2). These results indicate that artesunate has a strong anti-Mtb effect that causes the inactivation of Mtb, as well as induces anti-Mtb action that effectively inhibits or impedes the growth/proliferation of Mtb compared with artemisinin.

### 3.3. Determination of Anti-Mtb Effect of Artesunate by the Ogawa Slant Medium Assay

**Figure 4:**
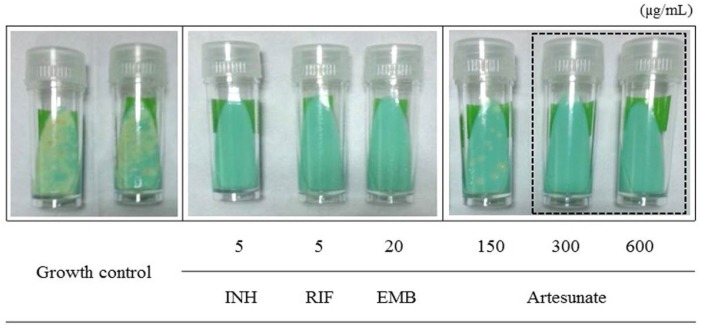
The anti-Mtb effect of artesunate against the growth/proliferation of Mtb confirmed using the Ogawa slant assay. Mtb was incubated with different concentrations (150–600 µg/mL) of artesunate on 2% Ogawa slant medium, respectively. Bacterial growth colonies were observed after three weeks of incubation. The dotted line indicates the anti-Mtb activity/effects of artesunate against Mtb. (INH; isoniazid, RIF; rifampicin, EMB; ethambutol). The Ogawa slant medium assay was carried out three times independently.

The anti-Mtb effect of artesunate was further measured using the Ogawa slant medium assay. In this assay, after Mtb was incubated with different concentrations (150–600 µg/mL) of artesunate for seven days, it was inoculated on the Ogawa slant medium, and its growth units were determined after three weeks. When Mtb was incubated with 300 µg/mL of artesunate, its growth colonies were not observed or proliferated on the Ogawa slant medium, and all anti-Mtb first-line drugs strongly inhibited the growth of Mtb (Figure 4). Particularly, the differences in drug susceptibility of Mtb were clearly confirmed when Mtb was incubated with 150 and 300 µg/mL of artesunate for 21 days with a single treatment. Furthermore, artesunate obviously showed its anti-tubercular activity/effect on the Ogawa slant medium by consistently inhibiting the growth of Mtb, and its minimum inhibitory concentration (MIC) was measured as 300 µg/mL. This result exhibits a similar tendency to that of the MGIT 960 system assay. Therefore, these results demonstrate that artesunate induces obvious anti-Mtb activity/effects in in vitro assay by effectively inhibiting or blocking the growth/proliferation of Mtb in a concentration-dependent manner. 

### 3.4. The In Vivo Anti-Mtb Effects of Artesunate and Artemisinin against M. tuberculosis

**Figure 5:**
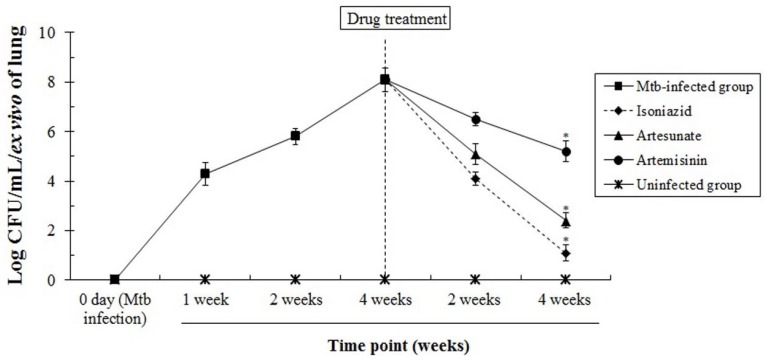
The in vivo tests of artesunate and artemisinin. The rats (six weeks/female) were divided into a normal group (*n* = 5), positive control group (*n* = 5/isoniazid group) and experimental groups (artesunate and artemisinin groups, *n* = 5/each group). The rats were orally administered with a single dose of 5 mg/kg of isoniazid, and 3.5 mg/kg of artesunate and artemisinin, once a day for four weeks, after four weeks of Mtb infection, respectively. The rats were carefully observed during the experimental periods. * *p* < 0.05 was considered to be statistically significant.

To evaluate the anti-Mtb effects of artesunate and artemisinin, their pharmacological effects were evaluated through an in vivo test using six-week-old female rats. The experimental groups were orally administered with a single dose of 3.5 mg/kg of the compound, once a day for four weeks, after four weeks of Mtb infection, respectively. After the extraction of the lungs from the rats, the number of Mtb was measured on 7H11 agar at each time point, and Mtb-growth units of Mtb-infected groups were not decreased or inhibited compared with drug-treated groups. Particularly, the growth/proliferation colonies of Mtb were significantly inhibited or decreased in the artesunate-treated groups compared with artemisinin-treated groups (Figure 5). These results demonstrate that artesunate not only induces a strong anti-Mtb effect/action by consistently inhibiting or blocking the growth/proliferation of Mtb compared with artemisinin in vivo, but also has potential to be used as an anti-Mtb agent through the selective Mtb-inhibitory ability. 

### 3.5. The In Vivo Toxicity Tests of Artesunate and Artemisinin

**Figure 6:**
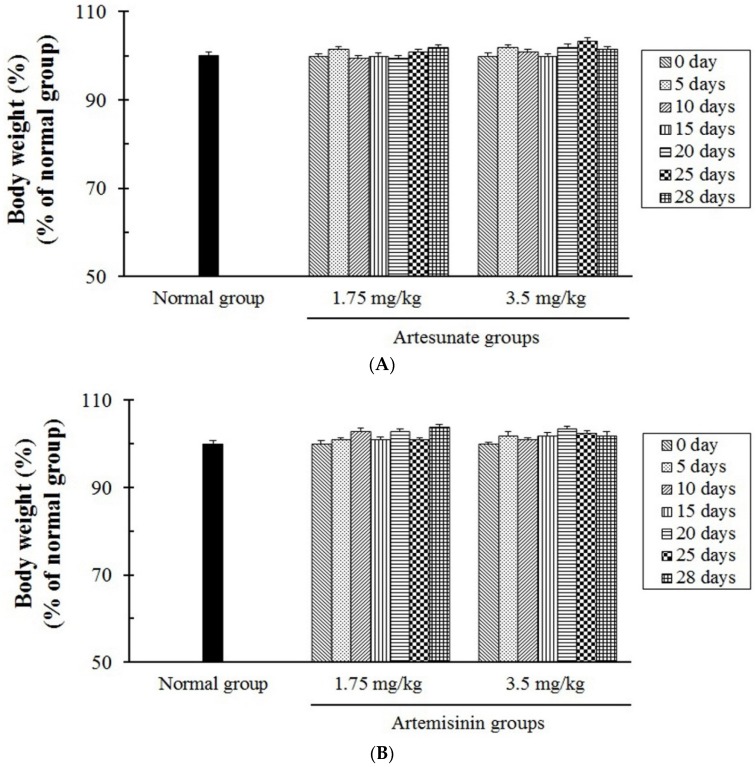
Changes of the body weight in rats following administration of artemisinin and artesunate. (**A**) The groups administered artesunate; and (**B**) the groups administered artemisinin. The rats were divided into a normal group (*n* = 4) and experimental groups (*n* = 8/artesunate two groups, *n* = 8/artemisinin two groups, *n* = 4/each group). The rats were orally administered with two different doses (1.75 and 3.5 mg/kg) of artemisinin and artesunate, once a day for four weeks, respectively, and additionally observed for seven days. The results were expressed as a percentage of the normal group and presented as the mean ± S.D.

To evaluate the toxicity of artesunate and artemisinin, the in vivo toxicity tests of artesunate and artemisinin were further evaluated using six-week-old female rats. The experimental groups were orally administered with two different doses (1.75 and 3.5 mg/kg) of the compounds, once a day for four weeks, respectively, and were additionally observed for seven days. In addition, the side effects and toxicity caused by the compounds were carefully observed in all the groups for the experimental periods. However, major symptoms of the side effects, such as the loss of body weight, vomiting, and diarrhea as well as the toxicity induced by the compounds, were not indicated or caused in all the experimental groups (Figure 6 and Table 3), and the animals treated by the compounds indicated the same survival rates and activity as the normal group during the experimental periods. Furthermore, artesunate showed a strong anti-Mtb effect/action compared with artemisinin, and there were no significant differences of the body weights between the experimental and normal groups. Therefore, these results obviously show that the compounds do not induce or cause toxicity and side effects when they indicate selective anti-Mtb activities/effects, or inhibit the growth/proliferation of Mtb through their novel pharmacological effects/action in in vivo tests.

## 4. Discussion 

Currently, various infectious diseases such as tuberculosis, Influenza, Severe acute respiratory syndrome (SARS), Middle east respiratory syndrome (MERS), Zika, and Ebola have caused serious concerns in public health fields worldwide. Tuberculosis, among these infectious diseases, is one of the most dangerous infectious factors, and it was responsible for the most deaths of the world’s population in 2014 and 2015 [1,2]. The increase of HIV co-infection and the rapid appearance of MDR or XDR strains represent an urgent need to develop novel, effective, and safe anti-Mtb drugs for treating TB. For the past few decades, in spite of various efforts to find new anti-Mtb drugs, the development of effective anti-Mtb drugs has still faced great difficulties and challenges. Some anti-tuberculosis drugs, such as streptomycin, rifamycin, and cycloserine, were derived from microorganisms for the development of novel anti-Mtb drugs and in order to block a serious public health threat, and they effectively induced direct pharmacological actions with secondary metabolites and novel mechanisms of action against tuberculosis [17,18,19,20]. The new pharmacological activities or functions of existing drugs used for treating different diseases can provide both safety and efficacy for rapid clinical trials compared with newly developed drugs, which may complement or predict side effects of drugs based on long-term accumulated clinical data. Furthermore, it is required to create effective strategies for the utilization of existing drugs as an alternative for developing effective/safe next-generation drugs, and as one of the feasible solutions for developing novel drugs against global challenges such as TB and MDR-TB or XDR-TB, as well as different intractable diseases. Furthermore, the compounds derived from the repurposing of existing drugs can improve or increase the potential for developing drugs for the next generation, and they can also serve as a useful resource for developing new drugs in the medical field. 

From these aspects, this study was focused on two key points for the discovery of new candidate substances as one of the various solutions for developing anti-Mtb drugs: first, the minimization of side effects and the safety of drugs, and second, finding novel pharmacological activity/effects of drugs causing anti-Mtb activity/effects through existing drugs which are used in other diseases. From these perspectives, new pharmacological activities/actions of artemisinin and artesunate have been consistently reported since the development of anti-malarial drugs, as follows: (1) the anti-viral activities of artesunate against human herpes viruses such as human cytomegalovirus (HCMV), herpes simplex virus 1 (HSV-1), human herpesvirus 6A (HHC-6a), and Epstein-Barr virus [21,22,23], as well as the inhibitory effect against hepatitis B virus (HBV) [24]; (2) the anti-parasitic effects of artesunate and artemisinin against the proliferation of *Toxoplasma gondii* [25,26,27] and the eggs of the liver flukes in an animal model [28,29]; (3) the anti-fungal activity of artemisinin against fungi, such as *Cryptococcus neoformans* and *Aspergillus fumigates* [30,31]; (4) the anti-arthritic effects of artesunate and artemisinin against rheumatoid arthritis through multi-signaling pathways in animal models [32,33,34]; (5) the anti-allergic activity of artesunate against allergic dermatitis in rat/guinea pig models [35,36,37]; (6) the anti-bacterial synergistic effects by a combination treatment of artesunate with ampicillin or oxacillin against sepsis animal models induced by *Escherichia coli* and methicillin-resistant *staphylococcus aureus* (MRSA) [38,39]; (7) the anti-cancer effects of artemisinin, which are caused by inducing or promoting apoptosis, and by inhibiting the growth of various cancer cells such as breast, colon, leukemia, and prostate cancer cells [40,41,42,43]; (8) the anti-allergic asthma effect of artesunate in a mouse model of allergic asthma [44]. These studies have demonstrated that artesunate and artemisinin have various biological effects/pharmacological actions, as well as the potential to be used as other therapeutic agents. However, in spite of their new pharmacological activities/actions provided through these studies, their anti-tuberculosis effects have not yet been reported or studied. Recently it was reported that compounds such as dihydroartemisinin-fluoroquinolone, synthesized using both artemisinin and fluoroquinolone, induced selective anti-tuberculosis activity [45].

From these perspectives, the results of this study showed the novel anti-Mtb ability/activities of artesunate and artemisinin by consistently inhibiting or blocking the growth/proliferation of Mtb. Particularly, the anti-Mtb activity/effects of artesunate were effectively demonstrated through selective anti-Mtb indicator assays such as the REMA, MGIT 960 system, and Ogawa slant medium assay, whereas the anti-Mtb effect of artemisinin was markedly low in activity compared with that of artesunate. In addition, artesunate showed its anti-Mtb activity and persistent effects by strongly inhibiting the growth/proliferation of Mtb for 21 days with a single treatment. In general, the cell wall of Gram-positive bacteria consists of only a single-unit-thick peptidoglycan layer and a plasma membrane. Furthermore, the mycobacterial cell wall is composed of a multi-complex of heteropolymers such as peptidoglycans, arabinogalactans, mycolic acids, and capsular lipids. In this respect, the differences in the specificity and superiority of artesunate and artemisinin regarding anti-Mtb activity/effects were confirmed by sensitive anti-Mtb assays such as the MGIT 960 system and the Ogawa slant medium assay, and may be associated with the susceptibility of Mtb caused by the interaction between various biomarkers, including crucial proteins regulating metabolic mechanisms in the cell membrane system or cell cycle key proteins that cause biochemical reactions in proliferation stages of Mtb, as well as the specificity between the different methods to detect or to evaluate anti-Mtb activity/effects. The important aspects that can be considered at this point in time are that the intracellular signaling pathways of replication and the cell cycle key proteins in the cytoplasm may be blocked or deactivated by the compound at check points that accelerate the growth/proliferation of Mtb, which suggests that the energy metabolism of the plasma membrane as well as biosynthesis related to the multi-complex, biochemical reactions and/or functions of the cell wall can be inactivated or inhibited through binding of the compound and Mtb. 

Particularly, artesunate in in vivo tests strongly showed anti-Mtb effects/action compared with artemisinin, which means that artesunate received less influence from the first-pass metabolism of the hepatic portal vein than artemisinin, and that the bioavailability of artesunate was more effective compared with that of artemisinin. In addition, it suggests that artesunate may act more effectively compared to artemisinin in the blood. Interestingly, this study showed that artesunate and artemisinin did not induce side effects and toxicity in in vivo tests, and that the compound-treated groups consistently induced the inhibition and decrease of Mtb, such as the isoniazid-treated group in in vivo tests. In addition, there were no differences in the survival rates in all groups. The results demonstrate that the compounds do not cause toxicity and side effects in in vivo tests when they indicate selective anti-Mtb activity/effects or inhibit the growth/proliferation of Mtb through their novel pharmacological actions/effects. 

In summary, artesunate and artemisinin effectively inhibited the growth/proliferation of Mtb, which can cause risks of severe complications and tuberculosis in patients with weakened immune systems, which showed their anti-Mtb activity/effects. In particular, artesunate strongly inhibited the growth of Mtb in a concentration-dependent manner compared with artemisinin. Furthermore, the anti-Mtb activities/effects of artesunate and artemisinin were demonstrated through different anti-Mtb indicator assays, such as in the REMA, MGIT 960 system and Ogawa slant medium assay. In particular, artesunate strongly showed new anti-Mtb effects/actions and its pharmacological effects compared with those of artemisinin in in vivo tests. Furthermore, the animals treated with artesunate and artemisinin showed vitality and/or activity comparable to the normal group in in vivo toxicity tests, which did not indicate toxicity and side effects such as the loss of body weight, vomiting, and diarrhea. The results of this study not only indicated the potential and/or the advantages for the effective utilization of artesunate and artemisinin in medical fields, but also demonstrated novel pharmacological activity/effects and biological actions/functions regarding the anti-Mtb activity/effects. Besides, these results indicate that artesunate has a higher potential for being repurposed through novel anti-Mtb activity/effects than artemisinin. 

Therefore, this study provides novel insights and the potential that artesunate can be utilized as a promising candidate agent of novel anti-TB drugs for the effective/safe next generation for the treatment of tuberculosis through its novel pharmacological activity/effects, and suggests its effective use as a potent Mtb inhibitor that can block or inhibit, beforehand, the prevalence and incidence rates of drug-resistant Mtb. In addition, artesunate may contribute to synergistic effects through combination therapy with standard drugs at the earliest stages of the disease, which needs further study regarding the effects and the safety through animal studies and clinical trials in the near future.

## Figures and Tables

**Table 1:** The anti-Mtb activity of artesunate and artemisinin against Mtb H37Rv measured by the REMA assay.

The Tested Compounds	Structure	Mol. Weight (g/M)	Concentrations (µg/mL) ^1^				
			300	150	75	37.5	18.75
Artesunate	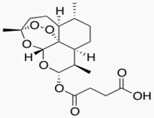	384.42	+	+	+	−	−
Artemisinin	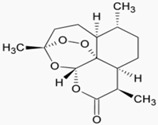	282.33	+	+	+	−	−

^1^ The anti-Mtb activity of the compounds against the growth of Mtb H37Rv was measured using the REMA assay. Mtb H37Rv was incubated with different concentrations (9.375–300 µg/mL) of the compounds at 37 °C for five days, respectively. The “+” sign shows anti-Mtb activity of the compounds against Mtb H37Rv. The results were carried out three times independently.

**Table 2:** Minimum inhibitory concentration (MIC) values of artesunate and artemisinin against the growth of Mtb H37Rv for different incubation periods.

Different Assays ^1^	Incubation Days	MIC (µg/mL) of the Compounds against Mtb H37Rv Growth	
		Artesunate	Artemisinin
REMA	5	75	75
MGIT 960 system	21	300	>600
Ogawa slant medium	21	300	>600

^1^ The anti-Mtb activity/effect of the compounds was measured by the REMA, MGIT 960 system, and Ogawa slant medium assay. Mtb H37Rv was incubated with different concentrations (9.375–300 µg/mL) of the compounds at 37 °C, respectively, and their susceptibility was confirmed by different anti-Mtb–indicator assays. The results were carried out three times independently.

**Table 3:** The results of in vivo toxicity test of artesunate and artemisinin.

The Tested Compounds	Side Effects of Rats Treated with Artesunate and Artemisinin, Respectively		
	The Loss of Body Weight	Vomiting	Diarrhea
Artesunate	−	−	−
Artemisinin	−	−	−

Note: Rats were orally administered with two different doses (1.75 and 3.5 mg/kg) of artesunate and artemisinin, once a day for the four weeks of the experimental periods, respectively, and the rats were additionally observed for seven days. The rats were carefully observed during the experimental period of five weeks. The “−” sign indicates that there were no side effects in rats administered with the compounds.

## Abbreviations

The following abbreviations are used in this manuscript:

Mtb	*Mycobacterium tuberculosis*
TB	Tuberculosis
MDR-TB	Multidrug-resistant TB
XDR-TB	Extensively drug-resistant TB
REMA	Resazurin microtiter assay
DMSO	Dimethylsulfoxide
OADC	Oleic acid/albumin/dextrose/catalase
MIC	Minimum inhibitory concentration
